# The impact of moderate to severe osteoarthritis on the physical performance and quality of life: a cross-sectional study in Greek patients (PONOS study)

**DOI:** 10.1186/s12891-023-06770-7

**Published:** 2023-08-15

**Authors:** P. Savvari, I. Skiadas, S. A. Papadakis, V. Psychogios, O. D. Argyropoulou, A. P. Pastroudis, G. A. Skarpas, A. Tsoutsanis, A. Garofalakis, G. Katsifis, D. Boumpas, D. Menegas

**Affiliations:** 1 Internal Medicine Department Pfizer Hellas, Neo Psychiko, 243 Mesogeion Avenue, Athens, SA 15451 Greece; 2 2nd Orthopedic Department, KAT General Hospital of Attica, Athens, Greece; 3 5th Orthopedic Department, Asclepeion General Hospital, Athens, Greece; 4 Department of Pathophysiology, National and Kapodistrian University of Athens, Athens, Greece; 5 6th Orthopedic Department, Asclepeion General Hospital, Athens, Greece; 6 3rd Orthopedic Department for Sports Injuries and Regenerative Medicine, Mitera General Hospital, Athens, Greece; 7 6th Orthopedic Department Hygeia Hospital, Athens, Greece; 8 1st Orthopedic Department, Mitera General Hospital, Athens, Greece; 9 Rheumatology Department, Naval Hospital Athens, Athens, Greece; 10 4th Internal Medicine Department, Attikon University Hospital, Athens, Greece

**Keywords:** Hip osteoarthritis, Knee osteoarthritis, Health Related Quality of Life (HRQoL), Joint diseases, Musculoskeletal diseases

## Abstract

**Background:**

Osteoarthritis (OA) represents a leading cause of disability with limited data available for the Greek patients.

**Objectives:**

To evaluate the impact of moderate to severe symptomatic hip/knee OA under treatment on physical performance and quality of life.

**Methods:**

A non-interventional, cross-sectional, epidemiological study of patients with moderate/severe OA, recruited in a single visit from 9 expert sites in Athens, Greece. Assessments were based on commonly used outcome scales: the Hip disability and Osteoarthritis Outcome Score (HOOS), the Knee Injury and Osteoarthritis Outcome Score (KOOS) and the EuroQol-5-Dimensions 3-levels questionnaire (EQ-5D-3L).

**Results:**

One hundred sixty-four patients were included in the analysis. Most of the patients were females (78.7%), with a mean age of 70.5 ± 10.2 years. Comorbidities were reported by 87.2% of patients with hypertension being the most frequently reported (53.7%), followed by dyslipidemia (31.1%), obesity (24.4%) and diabetes mellitus (23.2%). Paracetamol was the most common treatment (96%), followed by NSAIDs (75%), opioids (50%) and locally applied medications (42.7%). Both hip and knee OA patients showed substantial deterioration in health-related quality of life (QoL) and health status as reflected by the HOOS/KOOS (Function in sport and recreation was the most impaired subscale, followed by Hip- or Knee-related QoL). The mean EQ-5D-3L index score was 0.396 ± 0.319 and the mean EQ-VAS score was 52.1 ± 1.9. When compared indirectly to the local population norms our OA population had worse QoL indices.

**Conclusion:**

Our findings suggest the functional disability and impaired QoL of Greek patients with moderate/severe hip/knee OA under treatment emphasizing the need for novel treatments that will reduce the burden of the disease.

**Supplementary Information:**

The online version contains supplementary material available at 10.1186/s12891-023-06770-7.

## Introduction

Osteoarthritis (OA) is a complex, slowly evolving multifactorial arthritis that affects many joints with the knee being the most frequently affected, followed by the hand and hip [1, 2]. The clinical features of OA include pain and stiffness, which lead to disability and loss of function with detrimental effects on patients' quality of life (QoL) [1, 2]. The prevalence of the disease increased globally by 113.25% (from 247.51 million in 1990 to 527.81 million in 2019) mainly due to aging and increased rates of obesity [3].

Multiple risk factors have been involved in the pathogenesis of OA such as obesity, older age and female gender, with obesity being the strongest and best-established risk factor [4, 5]*.* OA is also often accompanied by comorbidities (i.e., stroke, hypertension, peptic ulcer, anxiety, depression, diabetes) [6–9]*.*

Current management includes nonpharmacologic, pharmacologic and surgical interventions. Most of the patients use multiple regimens to alleviate symptoms and changes in medication categories mainly due to side effects, intolerance or non-response are quite common [10].

In Greece, limited data exist, mainly focusing on the prevalence of the disease [11]. The aim of our study was to quantify the pain and physical impairment experienced in a Greek population of confirmed, moderate to severe OA, that is resistant, intolerable, or ineligible for paracetamol and/or NSAIDs and/or opioids. In addition, we identified the social and clinical characteristics of this population, as well as the impact of the disease on patients’ QoL.

## Materials and methods

### Design and study population

The PONOS study (A4091091) was a non-interventional (NI), cross-sectional, epidemiological study. The protocol conformed to the ethical guidelines of the 1975 Declaration of Helsinki and was approved by the respective institutional review boards of all participating sites.

Patients ≥ 18 years of age with confirmed and symptomatic hip or knee OA, of moderate to severe grade on radiographic examination [Kellgren-Lawrence (KL) grade 3–4], for whom treatment with paracetamol and/or NSAIDs and/or an opioid was ineffective, not tolerated or inappropriate were recruited in a single visit between 29 April 2021 and 30 November 2021 from 9 hospital outpatient departments/hospital clinics in Athens, Greece. Written informed consent was obtained from all patients. The rationale behind the enrollment criteria was to include a population with clinically advanced OA, as denoted by not being treatment naïve. In addition, an unmet need in terms of treatment could be more easily identified -if existed- in patients who had already received multiple regimens either due to non-efficacy, intolerance or side effects.

### Assessments

The following variables were retrieved from the medical records of each patient: demographic data, clinical characteristics, comorbidities and medications. Presentation of results regarding comorbidities and medications focused on predefined diseases and medications of interest.

Furthermore, each patient completed the following patient reported outcome measures (PROs): the Hip disability and Osteoarthritis Outcome Score (HOOS), the Knee Injury and Osteoarthritis Outcome Score (KOOS) and the EuroQol-5-Dimensions 3-Levels questionnaire (EQ-5D-3L).

#### HOOS

The HOOS is a 40-item self-administered hip-specific questionnaire including five subscales: Pain, Symptoms, Activity limitations in daily living (ADL), Sports and Recreation Function (Sport/Rec) and Hip-related Quality of Life (QoL), with a score ranging from 0 indicating extreme hip problems to 100 indicating no hip problems [12].

#### KOOS

The KOOS is a 42-item self‐administered knee-specific questionnaire also including five subscales: Pain, Symptoms, Activity limitations in daily living (ADL), Sports and Recreation Function (Sport/Rec) and Knee‐related Quality of Life (QoL), with a score ranging from 0 indicating extreme knee problems to 100 indicating no knee problems [13].

The HOOS and KOOS scores were transformed to the Western Ontario and McMaster Universities Osteoarthritis Index (WOMAC) to allow comparisons with other studies utilizing this clinical tool. The procedure of data transformation for both questionnaires is described in the Supplementary material.

#### WOMAC

The WOMAC index scores for the three subscales (i.e., Pain, Stiffness and Function) range from 0 (no pain or disability) to 100 (the most severe pain and disability), with higher scores indicating more severe symptoms and disability [14]. Since there were no formal criteria for the classification of WOMAC scores for the purposes of this study “severe” osteoarthritis potentially requiring joint replacement (JR) was arbitrarily defined by a WOMAC index score of 39 or greater, as reported by Canadian researchers [15].

#### EQ-5D-3L

The EQ-5D-3L self-administered questionnaire assesses health-related QoL (HRQoL) in five dimensions (mobility, self-care, usual activities, pain/discomfort and anxiety/depression) and three response levels (no problems, some problems, extreme problems) [16]. Since there is no Greek value set, the UK value set [17] and the Greek population norms [18] were used in the present study to calculate the EQ-5D-3L index score. The UK EQ-5D-3L scores set ranges from –0.594 to 1, with negative values corresponding to states worse than death, 0 to states equivalent to death and 1 to perfect health. In general, higher scores indicate better HRQoL [17].

#### EQ VAS

The EuroQol Visual Analogue Scale (EQ VAS) records the respondent’s overall current health on a scale from 0 (worst health imaginable) to 100 (best health imaginable) and provides a quantitative measure of the patient’s perception of their overall health status [16].

### Statistical analysis

Descriptive statistics are presented overall and by index joint. Especially for the EQ-5D-3L, the 25th and 75th percentile are presented instead of min and max, in order to have comparable results with the Greek population norms [18].

The analysis did not involve any group comparisons or inferences. Accordingly, the convenience sample under study comprised eligible patients presenting at the sites as part of their standard care and recruited in a consecutive manner. Descriptive statistical analysis was performed for all study data. The analysis was not adjusted for any potential confounders.

Statistical analysis and generation of all tables and figures were performed using RStudio (RStudio Team (2020). RStudio: Integrated Development for R. RStudio, PBC, Boston, MA) and/or IBM SPSS v.25 (IBM Corp. Released 2017. IBM SPSS Statistics for Windows, Version 25.0. Armonk, NY: IBM Corp.).

## Results

### Patients’ characteristics

A total of 164 patients were enrolled in the PONOS study between 29 April 2021 and 30 November 2021 from 9 hospital outpatient departments/hospital clinics in Athens, Greece. The sites were instructed to enroll eligible patients consecutively to reduce enrollment bias.

We selected the major referral centers in the most densely populated region of the country. As a result of a feasibility process and to enhance the external validity of the results, 3 rheumatology outpatient clinics, 3 major private hospital orthopedic clinics and 3 major outpatient orthopedic clinics from the public health system were invited and accepted to participate. In addition, the sites were chosen based on the large number of OA patients they handle, the expertise of the Principal Investigators on OA management and their experience in clinical trials. Some of the patients were recruited by the hospitals’ own databases, which kept detailed medical records. Since this was a single visit study performed in the context of the standard of care- with no intervention- there were no patients that declined to participate.

A summary of key demographic characteristics of the patients in the overall study population and in the hip and knee OA subpopulations is presented in Table 1. In the overall study population, the majority were females (129/164; 78.7%) with a mean age of 70.5 ± 10.2 years. The mean BMI was 28.2 ± 4.9 kg/m^2^, classified as “overweight”. Ninety-two out of 164 patients (56.1%) had knee OA, 66 out of 164 patients (40.2%) had hip OA and 6 out of 164 patients (3.7%) had OA in both joints (hip and knee). The knee was indicated as the index joint, i.e., the joint with the most severe OA in 96 out of 164 patients (58.5%) and the hip was the index joint for the remaining 68 patients (41.5%). The mean time since OA diagnosis of the index joint was 6.1 ± 5.7 years (Table 1).

**Table 1:** Patients’ demographic characteristics

	**All patients**	**Patients by index joint**	
		**Hip**	**Knee**
**Patients,** n	164	68	96
**Gender,** n (%)			
Female	129 (78.7)	52 (76.5)	77 (80.2)
Male	35 (21.3)	16 (23.5)	19 (19.8)
**Age,** years			
Mean (SD)	70.5 (10.2)	70.8 (10.4)	70.2 (10.1)
**Height,** cm			
Mean (SD)	165.4 (8.9)	167.2 (9.1)	164.1 (8.7)
**Weight,** kg			
Mean (SD)	77.1 (15.1)	74.9 (16.0)	78.6 (14.2)
**BMI**, kg/m^2^			
Mean (SD)	28.2 (4.9)	26.7 (4.5)	29.2 (4.9)
**Joint with OA,** n (%)			
Hip	66 (40.2)	-	-
Knee	92 (56.1)	-	-
Both	6 (3.7)	-	-
**Index joint (most severe),** n (%)			
Hip	68 (41.5)	-	-
Knee	96 (58.5)	-	-
**Time since index joint OA diagnosis,** years			
Mean (SD)	6.1 (5.7)	4.1 (2.8)	7.4 (6.8)

*n* number of subjects, *SD* Standard Deviation, *BMI* Body Mass Index, *OA* Osteoarthritis

Both types of OA were more prevalent in females, with 76.5% (52/68) and 80.2% (77/96) of women suffering from hip OA and knee OA, respectively. The mean age of participants with hip or knee OA was quite similar, i.e., 70.8 ± 10.4 and 70.2 ± 10.1 years, respectively. Also, the mean BMI was 26.7 ± 4.5 and 29.2 ± 4.9 kg/m^2^, respectively. Furthermore, the mean time since OA diagnosis of the hip and the knee joint was 4.1 ± 2.8 and 7.4 ± 6.8 years, respectively (Table 1).

As shown in Table 2, most patients (143/164; 87.2%) had comorbidities, with hypertension being the most frequently reported (88/164; 53.7%), followed by dyslipidaemia (51/164; 31.1%), obesity (40/164; 24.4%) and diabetes mellitus (38/164; 23.2%).

**Table 2:** Comorbidities and medications of interest

**Comorbidities, n (%)**^a^	
**Presence of comorbidities**	143 (87.2)
Hypertension	88 (53.7)
Dyslipidemia	51 (31.1)
Obesity	40 (24.4)
Diabetes mellitus	38 (23.2)
Depression	11 (6.7)
Other	66 (40.2)
**Medications**	
Paracetamol, n (%)	157 (95.7)
Mean (SD) (Pills/month)	35.3 (24.5)
NSAIDs (systemic), n (%)	123 (75)
Mean (SD) (Pills/month)	16.0 (14.9)
Opioids, n (%)	83 (50.6)
Mean (SD) (Pills/month)	15.5 (20.7)
Intraarticular (IA) hyaluronic acid, n (%)	35 (21.3)
Mean (SD) (In 12 months)	1.7 (1.1)
Intraarticular (IA) steroid, n (%)	32 (19.5)
Mean (SD) (In 12 months)	1.5 (0.8)
Locally applied medications	70 (42.7)
NSAID, n (%)	28 (17.1)
Diclofenac, n (%)	18 (11)
Capsaicin, n (%)	17 (10.3)
Other, n (%)^b^	7 (4.3)
Stem cells (number/year)	3
Platelet Rich Plasma (number/year)	1
Anxiolytics/sedatives, n (%)	17 (10.3)
Mean (SD) (Pills/month)	14.1 (10.3)
Antidepressants, n (%)	11 (6.7)
Mean (SD) (Packages in 3 months)	5.0 (5.8)
Anticonvulsants, n (%)	3 (1.8)
Mean (SD) (Packages in 3 months)	12.0 (15.6)

*IA *Intraarticular, *n* Number of subjects,* N/A *Not applicable,* NSAIDs*  Non-steroidal anti-inflammatory drugs,* SD* Standard Deviation

^a^% calculated on the overall population (*N* = 164)

^b^Other locally applied medications include balsam oil, cooling gel, etoricoxib, naproxen and nimesulide

Patients in the overall study population had a significant burden of analgesic treatment, with paracetamol being the most used drug (157/164; 96%), followed by NSAIDs (systemic) (123/164; 75%) and opioids (83/164; 50%). Consumption of a mean 35.3 ± 24.5, 16.0 ± 14.9 and 15.5 ± 20.7 pills/month was reported for paracetamol, NSAIDs (systemic) and opioids, respectively. Seventy patients (70/164; 42.7%) presented using locally applied medications e.g., NSAID, capsaicin and diclofenac gels, whereas 67 patients (67/164; 41%) received intraarticular (steroid or hyaluronic acid) injections and four patients stem cell or platelet rich plasma (PRP) injectable therapies. In addition, consumption of a mean 5.0 ± 5.8 (reported by 11/164; 6.7% patients) and 14.1 ± 10.3 (reported by 17/164;10.3% patients) packages of antidepressants and anxiolytics/sedatives within 3 months was reported, respectively (Table 2).

### Quality of life

#### Hip-related QoL

**Fig. 1:**
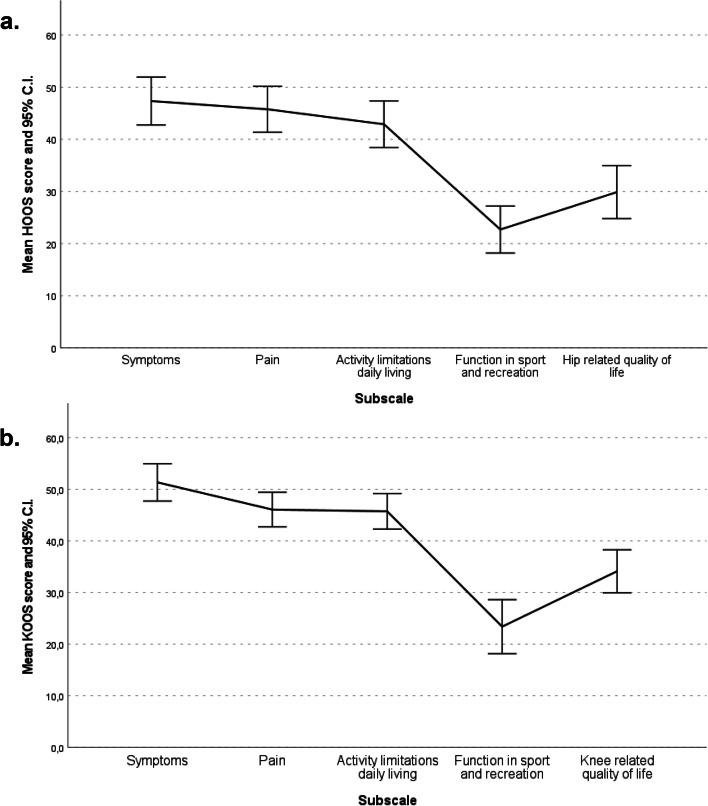
HOOS and KOOS profile. C.I. = Confidence Interval; HOOS = Hip dysfunction and Osteoarthritis Outcome score; KOOS = Knee injury and Osteoarthritis Outcome score

As shown in Table 3 and Fig. 1a, among patients with hip OA (*N* = 68), the HOOS subscale indicating the greatest impairment was that of Function in sport and recreation with a mean score of 22.7 ± 18.6, followed by the subscale Hip-related QoL with a mean score of 29.9 ± 21.0. On the other hand, the subscale indicating the least impairment was that of Symptoms with a mean score of 47.4 ± 19.0. Figure 1a illustrates the sample profile across the subscales.

**Table 3:** HOOS, KOOS questionnaire scores and corresponding WOMAC index scores

Variable	Mean (SD)	Range (min–max)	Floor (%)^a^	Ceiling (%)^a^
**HOOS subscales** *(n* = *68)*				
Symptoms	47.4 (19.0)	5.0–85.0	0.0	0.0
Pain	45.8 (18.2)	7.5–85.0	0.0	0.0
Activity limitations in daily living	42.9 (18.4)	8.8–89.7	0.0	0.0
Function in sport and recreation	22.7 (18.6)	0.0–75.0	13.2	0.0
Hip-related quality of life	29.9 (21.0)	0.0–81.2	4.4	0.0
**WOMAC subscales** *(n* = *68)*				
Pain	51.4 (19.7)	5.0–90.0	0.0	0.0
Stiffness	44.9 (24.2)	0.0–100.0	4.4	1.5
Function	42.9 (18.4)	8.8–89.7	0.0	0.0
**KOOS subscales** *(n* = *96****)***				
Symptoms	51.3 (17.8)	7.1–85.7	0.0	0.0
Pain	46.1 (16.5)	8.3–86.1	0.0	0.0
Activity limitations in daily living	45.7 (17.0)	8.8–89.7	0.0	0.0
Function in sport and recreation	23.4 (25.8)	0.0–100.0	27.1	2.1
Knee related quality of life	34.1 (20.5)	0.0–75.0	9.4	0.0
**WOMAC subscales** *(n* = *96)*				
Pain	48.5 (18.8)	5.0–95.0	0.0	0.0
Stiffness	49.9 (17.5)	12.5–87.5	0.0	0.0
Function	45.7 (17.0)	8.8–89.7	0.0	0.0

*HOOS* Hip dysfunction and Osteoarthritis Outcome score, *KOOS*  Knee injury and Osteoarthritis Outcome score, *n*  Number of subjects, *SD* Standard Deviation, *WOMAC* Western Ontario and McMaster Universities Osteoarthritis Index

^a^Floor is defined as the % of patients that have reached the lowest score (i.e., 0.0), while ceiling is defined as the % of patients that have reached the highest score (i.e., 100.0)

No patients reported the best possible score (ceiling effect) in any of the HOOS subscales, while the worst possible score (floor effect) was reported by 13.2% of the patients for the subscale Function in sport and recreation and by 4.4.% of patients for the subscale Hip-related QoL (Table 3).

Transformation of HOOS scores to WOMAC index scores revealed that WOMAC subscales had a mean score of 51.4 ± 19.7, 44.9 ± 24.2 and 42.9 ± 18.4 for Pain, Stiffness and Function, respectively, indicating severe pain, stiffness, and functional disability for the hip OA subpopulation. Only the subscale Stiffness showed floor and ceiling effects with 4.4% and 1.5% of patients reporting the worst and the best possible scores, respectively (Table 3).

### Knee-related QoL

As shown in Table 3 and Fig. 1b, among patients with knee OA (*N* = 96) the KOOS subscale indicating the greatest impairment was that of Function in sport and recreation with a mean score of 23.4 ± 25.8, followed by the subscale Knee-related QoL with a mean score of 34.1 ± 20.5, whereas the subscale indicating the least impairment was that of Symptoms with a mean score of 51.3 ± 17.8. Figure 1b illustrates the sample profile across all subscales. The best possible score (ceiling effect) was reported by 2.1% of patients for the subscale Function in sport and recreation, while the worst possible score (floor effect) was reported by 27.1% of patients for the subscale Function in sport and recreation and by 9.4% of the patients for the subscale Knee-related QoL.

The transformation of KOOS scores to WOMAC index scores revealed that WOMAC subscales had a mean score of 48.5 ± 18.8, 49.9 ± 17.5 and 45.7 ± 17.0 for Pain, Stiffness and Function, respectively, indicating severe pain, stiffness and functional disability for the knee OA subpopulation. There were no floor or ceiling effects observed (Table 3).

### Overall QoL

#### EQ-5D-3L questionnaire

The mean EQ-5D-3L index score was 0.396 ± 0.319, with most of the patients (118/164; 72%) having an EQ-5D-3L index score between 0–0.6. Only few patients (3/164; 1.8%) had a score between 0.8–1.0, corresponding to almost perfect health (Table 4).

**Table 4:** EQ-5D-3L questionnaire results

**EQ-5D-3L**	
n	164
Mean (SD)	0.396 (0.319)
Range (min–max)	-0.594–0.883
**EQ-5D-3L categories**, n (%)	
< 0.4	57 (34.8)
0.4–0.6	61 (37.2)
0.6–0.8	43 (26.2)
0.8–1.0	3 (1.8)

*EQ-5D-3L* EuroQol-5-Dimensions Questionnaire 3-levels questionnaire, *n* Number of subjects, *SD* Standard Deviation

Τhe frequencies of reported problems for each particular EQ-5D-3L dimension within the overall study population are presented in Table 5. The majority of patients reported having “some problems” in the EQ-5D-3L dimensions of mobility (142/164; 86.6%), self-care (109/164; 66.5%), usual activities (135/164; 82.3%) and pain/discomfort (123/164; 75.0%), while almost half of the patients reported having “some problems” in the dimension of anxiety/depression (71/164; 43.3%). The prevalence of reported problems for each EQ-5D-3L dimension within different age groups tended to increase with age, with a larger number of older patients exhibiting more problems in all EQ-5D-3L dimensions. Additionally, the proportion of patients reporting “no problems” in all dimensions (mobility, self-care, usual activities and anxiety/discomfort) except for pain/discomfort, tended to decrease with age.

**Table 5:** EQ-5D-3L problems reported by dimension (overall and by age group)

**Dimension**	**Total** *(n* = *164)*	**Age group** (years)			
		**44–54** *(n* = *13)*	**55–64** *(n* = *32)*	**64–75** *(n* = *61)*	**75 + ** *(n* = *58)*
	**n (%)**	**n (%)**	**n (%)**	**n (%)**	**n (%)**
**Mobility**					
No problems	20 (12.2)	3 (23.1)	4 (12.5)	6 (9.8)	7 (12.1)
Some problems	142 (86.6)	9 (69.2)	28 (87.5)	55 (90.2)	50 (86.2)
Confined to bed	2 (1.2)	1 (7.7)	0 (0.0)	0 (0.0)	1 (1.7)
**Self-care**					
No problems	50 (30.5)	5 (38.5)	12 (37.5)	20 (32.8)	13 (22.4)
Some problems	109 (66.5)	8 (61.5)	20 (62.5)	39 (63.9)	42 (72.4)
Unable to	5 (3.0)	0 (0.0)	0 (0.0)	2 (3.3)	3 (5.2)
**Usual activities**					
No problems	19 (11.6)	2 (15.4)	3 (9.4)	11 (18.0)	3 (5.2)
Some problems	135 (82.3)	10 (76.9)	29 (90.6)	47 (77.0)	49 (84.5)
Unable to	10 (6.1)	1 (7.7)	0 (0.0)	3 (4.9)	6 (10.3)
**Pain/Discomfort**					
No	5 (3.0)	0 (0.0)	2 (6.3)	1 (1.6)	2 (3.4)
Some	123 (75.0)	10 (76.9)	25 (78.1)	46 (75.4)	42 (72.4)
Extreme	36 (22.0)	3 (23.1)	5 (15.6)	14 (23.0)	14 (24.1
**Anxiety/Depression**					
No	60 (36.6)	7 (53.8)	17 (53.1)	21 (34.4)	15 (25.9)
Some	71 (43.3)	3 (23.1	9 (28.1)	27 (44.3)	32 (55.2)
Extreme	33 (20.1)	3 (23.1)	6 (18.8)	3 (4.9)	11 (19.0)

*EQ-5D-3L* EuroQol-5-Dimensions Questionnaire 3-levels questionnaire, *n* Number of subjects

### EQ-VAS scores

**Fig. 2:**
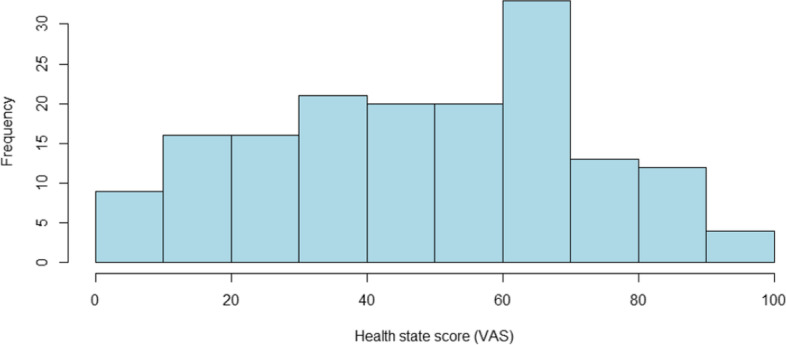
EQ-VAS frequency distribution (histogram). EQ VAS = EuroQol Visual Analogue Scale

Substantial deterioration in the self-perceived overall health status of patients with moderate to severe hip/knee OA as reflected by a mean EQ-VAS score of 52.1 ± 1.9 was reported in our study (Table 6). The EQ-VAS scores were left-skewed and responses were clustered predominantly around 60 and 70 on the EQ-VAS scale. Only approximately 15% of the patients rated their health status > 80 on the EQ-VAS scale (Fig. 2).

**Table 6:** EQ-VAS ratings (overall and by age group)

**EQ-VAS**	**Total**	**Age group** (years)			
		**45–54**	**55–64**	**65–74**	**75 + **
n	164	13	32	61	58
Mean (SE)	52.1 (1.9)	56.2 (6.8)	59.2 (4.1)	50.1 (3.4)	48.5 (2.7)
25^th^ percentile	33.8	40.0	40.0	30.0	36.3
75^th^ percentile	70.0	80.0	80.0	80.0	65.0

*EQ VAS *EuroQol Visual Analogue Scale*, n *number of subjects,* SE *Standard Error

Additionally, patient-reported overall health status tended to further deteriorate with age. Patients aged between 45–54 years had a mean EQ-VAS score of 56.2 ± 6.8, that was further decreased in patients between 65–74 and 75 + years old, with the mean EQ-VAS scores being 50.1 ± 3.4 and 48.5 ± 2.7, respectively (Table 6).

## Discussion

Our study is the first cross-sectional study, which quantified the physical impairment in a Greek population of confirmed, moderate to severe OA, resistant/intolerable or ineligible for paracetamol and/or NSAIDs and/or opioids. Our findings show that patients with OA experience significant pain/stiffness and functional disability, which mainly impairs their participation in sport/recreation activities. These restrictions along with anxiety and depression decrease their quality of life.

The profile of our OA patient is consistent with the typical profile of patients suffering from OA in daily clinical practice, i.e., mainly “overweight”, female of advanced age; a finding consistent with national and international reports (Greece [11]*;* Europe [8, 19, 20]; Middle East (Israel) [21]; Canada [22] and USA [23]).

In addition, our results point out that OA is not an isolated entity since it is frequently accompanied by comorbidities. In our study, most patients suffered from comorbidities with hypertension being the most frequently reported, followed by dyslipidemia, obesity and diabetes. These findings are also supported by the existing literature, which mentions that OA patients are 1.2 times more likely to have any comorbidity compared to non-OA and 2.5 times more likely to have ≥ 3 comorbidities [9]. More specifically, the most common comorbidities reported are cardiovascular (stroke, hypertension), gastrointestinal (peptic ulcer), psychiatric (anxiety, depression) and endocrine (diabetes, obesity) diseases [6–9, 20, 21, 23]*.* The clinical implication of this finding includes dilemmas in the disease management (eg. polypharmacy, drug interactions, higher rate of adverse effects), as well as increased economic burden [8, 9, 24].

In terms of pharmacological treatment existing evidence suggests limited efficacy accompanied by safety issues for both paracetamol [25–27] and opioids [28, 29]. In this context, some guidelines recommend either against the use [30] or short-term administration of paracetamol/opioids [31, 32]. Opioids are recommended as the last option for the severely symptomatic patient before surgery [31, 33].

Having said that, our data revealed an increased use of paracetamol and opioids with the majority of OA patients receiving paracetamol (96%) and half of them opioids (50%). This finding could be indirectly compared to other European countries, which report quite variable use of paracetamol (0–75.5%) and opioids (1.8% to 54.5%) [19, 20, 34, 35]. However, any comparison made should take into account the differences in the OA population included in these studies, country-specific factors in drug supply and availability, as well as variations in national treatment guidelines.

The present study allowed us to investigate how painful and restrictive moderate to severe hip or knee OA is using the HOOS and the KOOS questionnaires, respectively. According to our results, both hip and knee OA patients showed similar subscale pattern of impairment (i.e., most impaired subscale Function in sport and recreation, followed by Hip- or Knee-related QoL), consistent with that reported in other European studies [36–40] of hip/knee OA prior to total JR*.* The worst possible score for the aforementioned subscales was also comparable in numbers between our study and other studies conducted in patients with advanced disease [36, 38–40]. Severe stiffness and functional impairment were also indicated by the WOMAC index scores in all subscales.

The physical disability was further captured in the diminished quality of life as indicated in the EQ-5D-3L questionnaire by almost two third of our patients. A similar EQ-5D-3L index score was reported either among preoperative knee OA patients [41] or in patients with hip or knee OA, but without specified disease severity (i.e., mild, moderate or severe)) [42]. Furthermore, when our population was compared to the local population norms a trend of reporting having “some problems” across all EQ-5D-3L dimensions that increased with age was evident, contrary to the Greek norms that reported having “no problems” across all EQ-5D-3L dimensions [18]. This shift was particularly evident for the Self-care and Usual activities, with approximately seven- and four-fold increase in the proportion of patients, respectively, compared to the norms [18]. An even higher increase was evident in the “extreme problems” level for the Usual activities and Anxiety/Depression dimensions, with twelve- and six-fold increase, respectively, compared to the norms [18]. Of note, disease associated anxiety was particularly disturbing for the younger age group (44–54 years old), since a higher proportion of patients reported “extreme” problems compared to the norms [18].

Substantial deterioration in the self-perceived overall health status of patients with moderate to severe hip/knee OA was further confirmed by EQ-VAS score in which only 15% rated their health status as substantially “good”, reflecting the significant l burden imposed by the disease. Not surprisingly, the mean EQ-VAS score found in our study was considerably lower (52.1 vs 79.0) than the respective score of the Greek norms in all age groups, as reported by Yfantopoulos [18]. This finding highlights the unmet need for novel treatments that could provide symptomatic relief, as well as modify the progression of the disease.

Overall, the PONOS study suggests that our population of moderate/severe OA under treatment suffers from severe pain, stiffness, and disability. This is reflected in the reduced self-perceived HRQoL, leading to loss of autonomy and impairment of social relationships and psychological well-being.

### Limitations

Our study due to its design was descriptive with no control group. It was conducted in one region of the country and mainly in an urban population, thus the generalization of the results to the overall population are subject to limitations. In addition, our sample of moderate/severe OA patients is not representative of the general OA population. Although participants were recruited consecutively, selection bias cannot be dismissed.

Moreover, although PROs are designed to reduce recall bias, over or underestimation of differences in specific self-reported patients’ characteristics and questionnaires scores are possible. In terms of medication use, our results were also based on PROs and physicians’ records and not on more detailed methods eg. medication possession ratio. On the other hand, the study’s strength lies in the fact that it included a well-defined, relatively large, expertly assessed group of patients with moderate to severe hip and knee OA under treatment, which was assessed with valid questionnaires. Our study is the first which offers real world data for the Greek population with moderate/severe OA resistant/intolerable or ineligible to treatment.

## Conclusions

OA has become one of our most important public health problems—a problem that is expected to worsen in the following decades given the aging and escalating levels of obesity. Despite several available treatment options and guidelines for the management of symptoms, patients continue to suffer from chronic pain, leading to physical inactivity, loss of autonomy, social distancing and compromised QoL. The PONOS study highlighted for the first time in Greece, both the functional disability and impaired QoL of this OA patient population. The results of our study highlight the unmet medical need for innovative treatment options and emphasize the need for appropriate intervention through public health strategies aiming to address risk factors and timely diagnose the disease.

### Supplementary Information

**Additional file 1.** 

## Data Availability

The data that support the findings of this study are available from Pfizer Hellas, but restrictions apply to the availability of these data, which were used under license for the current study, and so are not publicly available. Data are however available from the corresponding author upon reasonable request and with permission of Pfizer.

## Abbreviations

ADL: Activity limitations in Daily Living
BMI: Body Mass Index
EQ-5D-3L: EuroQol-5-Dimensions 3-Levels questionnaire
EQ VAS: EuroQol Visual Analogue Scale
HOOS: Hip dysfunction and Osteoarthritis Outcome Score
HRQoL: Health-Related Quality of Life
IA: Intraarticular
JR: Joint Replacement
KL: Kellgren-Lawrence
KOOS: Knee injury and Osteoarthritis Outcome Score
NI: Non-Interventional
NSAID: Non-steroidal anti-inflammatory drug
OA: Osteoarthritis
OARSI: Osteoarthritis Research Society International
PRP: Platelet Rich Plasma
QoL: Quality of Life
SD: Standard Deviation
SE: Standard Error
WOMAC: Western Ontario and McMaster Universities Osteoarthritis Index
PROs: Patient Reported Outcomes
