# Supplementary material for: The impact of moderate to severe osteoarthritis on the physical performance and quality of life: a cross-sectional study in Greek patients (PONOS study)
Source: BMC Musculoskelet Disord. 2023 Aug 15;24:651. doi: 10.1186/s12891-023-06770-7 (PMC10426090; doi:10.1186/s12891-023-06770-7)
Supplement: Supplementary file 1 — Additional file 1. [file 12891_2023_6770_MOESM1_ESM.docx]

**Supplementary material**

**Data transformation**

In the present study the following variables were also calculated using the formulas and algorithms presented below.

- ***WOMAC index transformation formulas***

The HOOS and KOOS scores were transformed to the WOMAC index.

In order to get the original WOMAC scores, the item scores for each HOOS subscale had to be summed. Additionally, for percentage scores in accordance with the HOOS, the formula provided below had to be used to convert to the original WOMAC scores.

Transformed scale = 100 - actual raw score x 100

Possible raw score range

| WOMAC subscores | Original score = sum of the following items | Possible raw score range |
| --- | --- | --- |
| Pain | P4-P8 | 20 |
| Stiffness | S4-S5 | 8 |
| Function | A1-A17 | 68 |

In order to get the original WOMAC Scores, the item scores for each KOOS subscale had to be summed. Additionally, for percentage scores in accordance with the KOOS, the formula provided below had to be used to convert to the original WOMAC scores.

Transformed scale = 100 - actual raw score x 100

maximum score

| WOMAC subscores | Original score = sum of the following items | Maximum score |
| --- | --- | --- |
| Pain | P5-P9 | 20 |
| Stiffness | S6-S7 | 8 |
| Function | A1-A17 | 68 |

- ***EQ-5D-3L algorithm***

The 5-digit state derived from the EQ-5D-3L instrument was transformed into an index value by attaching weights to the levels of each dimension. The set of the weights used is called the ‘value set’. Since there is no Greek value set, the UK value set and the Greek population norm was used, as described in Yfantopoulos (Yfantopoulos Y 1999). The following algorithm was applied to calculate the EQ-5D-3L Total Score:

1. If **all** five EQ-5D dimensions have a score of 1 then the EQ-5D-3L Total Score is 1.
2. If **any** of the five EQ-5D dimensions has a score of 3, then the EQ-5D-3L Total Score is:

$$1-0.081-\left( \sum_{1}^{5} weighted dimension score \right)-0.269$$

3. If **none** of the five EQ-5D dimensions has a score of 3, then the EQ-5D Total Score is:

$$1-0.081-\left( \sum_{1}^{5} weighted dimension score \right)$$

The EQ-5D-3L weightings are presented below (from Dolan et al 1995):

| **EQ-5D Dimension** | **Score = 1** | **Score = 2** | **Score = 3** |
| --- | --- | --- | --- |
| Mobility | 0 | 0.069 | 0.314 |
| Self-Care | 0 | 0.104 | 0.214 |
| Usual Activities | 0 | 0.036 | 0.094 |
| Pain/Discomfort | 0 | 0.123 | 0.386 |
| Anxiety/Depression | 0 | 0.071 | 0.236 |

**Supplementary References**

Yfantopoulos Y. Quality of life measurement and health production in Greece. In: Greiner W, Schulenburg J-M. Graf v.d., Piercy J (eds) (EuroQol) Plenary meeting. Discussion papers. Uni-Verlag Witte, Hannover, 1999, pp 100–114.

Dolan P, Gudex C, Kind P and Williams A. 1995. A social tariff for EuroQol: results from a UK general population survey. Working Papers 138chedp, Centre for Health Economics, University of York.
